# Can Data-Driven Supervised Machine Learning Approaches Applied to Infrared Thermal Imaging Data Estimate Muscular Activity and Fatigue?

**DOI:** 10.3390/s23020832

**Published:** 2023-01-11

**Authors:** David Perpetuini, Damiano Formenti, Daniela Cardone, Athos Trecroci, Alessio Rossi, Andrea Di Credico, Giampiero Merati, Giampietro Alberti, Angela Di Baldassarre, Arcangelo Merla

**Affiliations:** 1 Department of Neurosciences, Imaging and Clinical Sciences, University “G. d’Annunzio” of Chieti-Pescara, 66100 Chieti, Italy; 2 Department of Biotechnology and Life Sciences (DBSV), University of Insubria, Via Dunant, 3, 21100 Varese, Italy; 3 Department of Engineering and Geology, University “G. d’Annunzio” of Chieti-Pescara, 65127 Pescara, Italy; 4 Department of Biomedical Sciences for Health, University of Milan, 20129 Milan, Italy; 5 Department of Computer Science, University of Pisa, 56127 Pisa, Italy; 6 Department of Medicine and Aging Sciences, University “G. d’Annunzio” of Chieti-Pescara, 66100 Chieti, Italy; 7 IRCCS Fondazione Don Carlo Gnocchi, 20148 Milano, Italy; 8 University of Milan, 20122 Milan, Italy

**Keywords:** electromyography (EMG), muscular fatigue, muscular activity, thermography, machine learning (ML)

## Abstract

Surface electromyography (sEMG) is the acquisition, from the skin, of the electrical signal produced by muscle activation. Usually, sEMG is measured through electrodes with electrolytic gel, which often causes skin irritation. Capacitive contactless electrodes have been developed to overcome this limitation. However, contactless EMG devices are still sensitive to motion artifacts and often not comfortable for long monitoring. In this study, a non-invasive contactless method to estimate parameters indicative of muscular activity and fatigue, as they are assessed by EMG, through infrared thermal imaging (IRI) and cross-validated machine learning (ML) approaches is described. Particularly, 10 healthy participants underwent five series of bodyweight squats until exhaustion interspersed by 1 min of rest. During exercising, the vastus medialis activity and its temperature were measured through sEMG and IRI, respectively. The EMG average rectified value (ARV) and the median frequency of the power spectral density (MDF) of each series were estimated through several ML approaches applied to IRI features, obtaining good estimation performances (*r* = 0.886, *p* < 0.001 for ARV, and *r* = 0.661, *p* < 0.001 for MDF). Although EMG and IRI measure physiological processes of a different nature and are not interchangeable, these results suggest a potential link between skin temperature and muscle activity and fatigue, fostering the employment of contactless methods to deliver metrics of muscular activity in a non-invasive and comfortable manner in sports and clinical applications.

## 1. Introduction

Electromyography (EMG) is the measurement of the electrical signals produced by activated muscles [1]. When a muscle fiber is stimulated by a motor neuron, the ionic concentration surrounding the semi-permeable lipid membrane changes, resulting in the formation of a biopotential gradient. The EMG sensors detect the simultaneous occurrence of these potentials elicited by muscular activation as the sum of the motor unit action potential. This technique is widely used to investigate possible pathologic changes [2] or rehabilitation benefits, as well as to evaluate neuromuscular responses in sports applications. For example, EMG is used to evaluate muscle activity under a variety of running conditions (e.g., on the ground or on a treadmill, at varying speeds and on different surfaces, and during resistance training and weightlifting) [3].

Time-, frequency-, and non-linear-domain approaches are typically utilized to analyze the EMG signal to obtain metrics indicative of muscular activity. Typically, time-domain metrics are used to detect muscle force [4]. The average rectified value (ARV) is one of these metrics. It represents the area under the normalized EMG signal divided by the considered temporal window [5,6,7]. The frequency-domain variables are calculated using the power spectral density (PSD). Specifically, the median frequency (MDF) of the PSD is utilized to detect muscle fatigue [2,7].

Additionally, novel techniques based on complexity analysis and machine learning (ML) have been applied to the analysis of EMG data (for a recent review, see Rampichini et al., 2020 [8]). So far, nonlinear analyses based on fractals and self-similarity (as fractal dimensions, detrended fluctuation analysis, multifractality), correlation (as correlation dimension and recurrence quantification analysis), entropy (as approximate, sample, and fuzzy entropies) and deterministic chaos (as Lyapunov Exponent) have been used to detect muscle fatigue in EMG signal [9]. Furthermore, classifications of sEMG patterns via k-means clustering or support vector machines [10,11] and the evaluation of gait disorders through several ML approaches [12] have been proposed.

EMG signals may be recorded in either a contact or contactless fashion [13]. The contact method could be implemented using a needle-like probe inserted invasively into the human muscle or electrodes capable of measuring EMG signals through the skin (surface EMG, sEMG). Compared to sEMG electrodes, invasive probes offer superior diagnostic performance and better sensitivity of muscle abnormalities [14]. However, the invasive method requires surgical preparation and trained operators; therefore, sEMG is preferred whenever possible. Typically, Ag–AgCl conductive gel is utilized to increase conductivity, improve electrode–skin coupling, and reduce motion artifacts. The primary disadvantage of this type of electrode is the need for skin preparation prior to measurement. In addition, the conductive gel may cause skin irritation and discomfort, and it dries out over time, rendering long measurements noisy [15]. Moreover, the use of an electrolyte for chronic use is inconvenient because the reliance on an electrolyte results in a decrease in signal quality as the gel dehydrates [16], and the reapplication of gel may not be possible. In addition, the recording may occur in a sensitive area, a previous skin treatment may render standard electrodes ineffective, or the required distance between electrodes may be so close that smearing of the electrolyte would occur [17,18]. Furthermore, the application and removal of electrolyte gels is unpleasant for the patient and time-consuming for the clinician or caregiver [19]. Finally, although rare, dermatological reactions can occur [16,17,18].

In contrast, dry electrodes do not require any conductive gel because sweat from the human body is used to improve contact. However, due to their high input impedance, dry electrodes are highly susceptible to motion artifacts. Although various innovative electrode designs have been proposed to improve the performance of dry electrodes [20,21], they are still highly susceptible to motion artifacts. To overcome these limitations, capacitive EMG sensors that do not require direct skin contact have been developed; however, they are still highly sensitive to motion artifacts and are not comfortable for prolonged monitoring.

The development of methods capable of estimating EMG parameters at a distance, without requiring skin-located sensors, could facilitate the use of EMG for long-term recordings. In this regard, it is well established in the scientific literature that the skin temperature above a muscle was found to be related to EMG muscular activity [22]. For example, Rodriguez-Sanz et al. discovered a significant correlation between EMG MDF and skin temperature after running (Pearson correlation coefficient *r* = 0.78) [23]. Moreover, Shakihih et al. discovered a statistically significant correlation between the average temperature and the root mean square (RMS) and the MDF of the sEMG signal during repeated calf raises [24]. In order to assess temporomandibular joint dysfunction in juvenile idiopathic arthritis, the EMG ratio between the masseter and temporalis muscles was estimated from skin temperature [25].

In this regard, infrared thermal imaging (IRI) could be a suitable method for noninvasively measuring skin temperature above any skeletal muscle. IRI is a technique that measures the radiant energy emitted by a body to estimate its surface temperature from a distance [26]. IRI is noninvasive, contactless, transportable, and inexpensive. This technology is extensively used in biomedical and athletic applications to monitor skin temperature for a variety of purposes (e.g., circulatory disease assessment, affective computing, and thermoregulation) [27]. Particularly, thermal imaging is frequently employed in sports science to assess the physical condition of athletes by measuring their muscle temperature during exercise and their perceived fatigue [28].

This study tested a non-invasive, contactless method for estimating parameters indicative of muscular activity and fatigue as it is assessed by EMG using IRI data. Specifically, cross-validated data-driven multivariate procedures were applied to features computed from the temperature time course of the skin above the activated musculature, to estimate the ARV and MDF as measured by sEMG. Notably, the aim of the study is not to measure the EMG signal from skin temperature variations assessed through IRI, but to find correlations between metrics evaluated from the EMG signal and IRI signal features, although the two processes are of a different physiological nature.

## 2. Materials and Methods

### 2.1. Participants

Ten active adults (6 males and 4 females) volunteered to take part in this study. Their mean age, body mass, and height were 21.8 ± 2.9 yrs, 70.9 ± 6.3 kg, 176.2 ± 6.3 cm (mean ± standard deviation, STD), respectively. They were habitually physically active, but none of them was used to resistance training. All participants were non-smokers, not injured, and without cardiovascular or pulmonary diseases. They had not consumed drugs or medications with a potential effect on cardiovascular and thermoregulatory functions during the two months before the tests. A week before the testing session, they removed body hair on thighs that were clean and without cosmetics products.

### 2.2. Experimental Protocol

**Figure 1:**
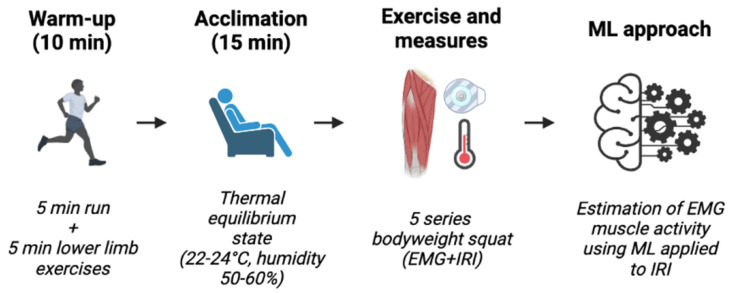
Schematic overview of the testing session. The figure is created with BioRender.com, EMG: electromyography; IRI: infrared thermal imaging; ML: machine learning.

After a preliminary session aimed at familiarizing them with the testing procedures, participants underwent a testing session in the following week. In the testing session, after a 10 min warm-up composed of 5 min of running and 5 min of dynamic lower-limb activities (such as low intensity jumping and lunging drills), participants acclimated to the room climate conditions (temperature 22–24 °C; relative humidity 50–60%; no direct ventilation and constant intensity of light) for 15 min at resting condition to achieve thermal equilibrium between the body and the environment [29]. Then, participants performed 5 sets of bodyweight squats until exhaustion (maintaining hands on the hips) interspersed by 1 min of rest, during which EMG and IRI were recorded simultaneously. In Figure 1, a representative figure of the testing session is reported. A strength and conditioning coach supervised the entire session, ensuring that participants maintained an appropriate movement quality throughout the experiment. The speed of movement was standardized with the aid of a metronome (1 s for eccentric, 1 s for relaxing, 1 s for concentric phase). The subjects were instructed to refrain from strenuous physical activity during the two days before the trials and abstained from assuming alcoholic or caffeine-containing products for a 4 h period before the start of the experiment. All sessions were scheduled in the late morning to mitigate possible effects related to circadian rhythm changes. The IRI measurements were performed in accordance with the guidelines provided by Moreira et al., 2017 [30].

### 2.3. Electromyography Recording and Preprocessing

EMG was recorded employing the Encephalan Mini AP-10 system. The sEMG sensor acquired at a sample frequency of 250 Hz. To maximize skin–electrode coupling, the adhesion areas of the electrodes were shaved and cleaned with 96% isopropyl alcohol and cotton. The bipolar sEMG Ag/AgCl electrodes (Ceracarta, Forlì, Italy) were positioned 2 cm apart along the muscle belly fibers in a longitudinal orientation. Particularly, they were positioned at 80% of the distance between the superior anterior iliac spine and the joint space at the anterior border of the medial collateral ligament on the left vastus medialis (VM) [31].

Concerning the sEMG processing, the signals were high-pass filtered (with a cut-off frequency of 20 Hz) with a zero-lag 3rd order Butterworth filter and rectified. The filtering cut-off frequency was chosen based on previous studies and guidelines [32,33,34]. Particularly, De Luca et al. found that for applications involving isometric contractions or natural and common movements (such as squats), the recommended corner frequency for a Butterworth filter is 20 Hz [35].

Then, the MDF, which is indicative of muscle fatigue, is the frequency at which the power spectrum of the EMG signal is divided into two parts of equal width, as reported below:

$$\overset{\mathrm{MDF}}{\underset{i=1}{\sum }}P_{i}=\overset{N}{\underset{i=\mathrm{MDF}}{\sum }}P_{i}=\frac{1}{2}\overset{N}{\underset{i=1}{\sum }}P_{j}$$

where *P_i_* is is the EMG power spectrum at the *i*th frequency bin, and *N* is the number of frequency bins. In this study, the power spectrum of the EMG signal was extracted through Welch’s method [36] and *N* was set at the next power of 2 from the length of EMG time-domain signal [37].

The ARV was computed to evaluate the muscular activity. It is defined as the averaging of the rectified EMG signal across the duration of a motor task [7]. It is computed as follows:

$$\mathrm{ARV}=\frac{1}{N}\overset{N}{\underset{n=1}{\sum }}\left|\mathrm{EMG}\left[n\right]\right|$$

where *N* is the number of samples to be averaged. Since a fatiguing exercise until exhaustion was employed in this study, the number of samples depended on the performance of the participants.

These metrics were evaluated for each series (5 series for each participant), resulting in 50 values for both ARV and MDF.

### 2.4. Infrared Thermal Imaging Recording and Preprocessing

Thermal infrared camera FLIR SC660 (FLIR, Wilsonville, OR, USA) (640 480 bolometer FPA, sensitivity/noise equivalent temperature difference: 30 mK at 30 °C, field of view: 24° 18°) was used to record the VM’s temperature. The camera was placed 60 cm away from the subject, focused on the subject’s legs, and acquired at a sample rate of 10 Hz.

To eliminate optical artifacts and the potential drift/shift in sensor response, the camera was blackbody-calibrated. The acquisitions were carried out in accordance with thermal measurement standards. In order to minimize thermoregulatory effects, IRI measurements were conducted in a temperature-neutral environment.

**Figure 2:**
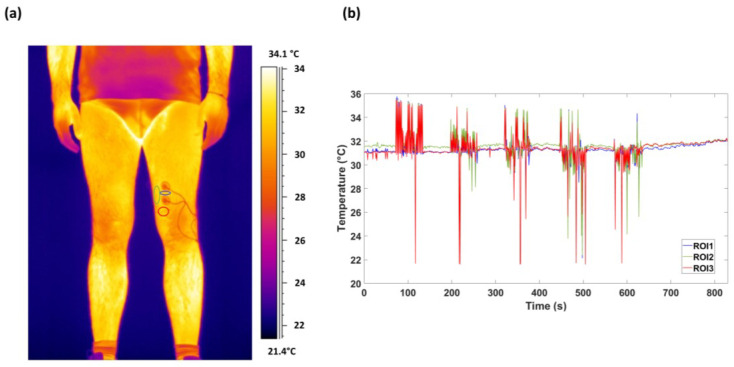
(**a**) A representative participant’s thermogram and ROI placement over the VM. Specifically, the blue, green, and red circles are the ROI1, ROI2, and ROI3, respectively. (**b**) The time course of the average temperature of each ROI during the experiment, obtained through a video tracking algorithm.

The quality of recordings was evaluated visually, and no video was discarded. As shown in Figure 2a, three regions of interest (ROIs) were chosen around the Ag/AgCl electrodes. Particularly, ROI1 was placed between the two electrodes, ROI2 laterally to the electrodes, and ROI3 below the electrodes. Notably, the ROIs were chosen in order to cover the VM muscle avoiding the influence of the electrodes on the recorded muscle temperature. A video tracking algorithm was utilized to track the ROI across thermal video frames [38]. During the experiment, a representative temperature time course is depicted in Figure 2b.

### 2.5. Features Extraction and Machine Learning Procedures

The features for the ML-based regression were computed on the selected ROIs after the end of each series. Specifically, the features extracted from the IRI signals are:

1. Mean value (*MeanTemp*): average value of the thermal signal *T* over a temporal window of 10 s after the end of the exercise defined as:
  $$MeanTemp=\frac{1}{N}\overset{N}{\underset{i=1}{\sum }}Ti$$
  where *N* is the number of samples.
2. Standard deviation (STD): standard deviation of the thermal signal *T* over a temporal window of 10 s after the end of the exercise defined as:
  $$\mathrm{STD}=\sqrt{\frac{1}{N-1}\overset{N}{\underset{i=1}{\sum }}{\left(T_{i}-MeanTemp\right)}^{2}}$$
  where *N* is the number of samples.
3. Mean value of the power spectral density (*MeanPSD*) of the thermal signal *T* over a temporal window of 10 s after the end of the exercise. The PSD is defined as the Fourier transform of the autocorrelation matrix $R_{x}\left(\tau \right)$ of a random process X(τ):
  $$S_{X}\left(f\right)=F\left\{R_{x}\left(\tau \right)\right\}=\int_{-\infty }^{+\infty }R_{x}\left(\tau \right)e^{-2j\pi f\tau }d\tau$$
4. Kurtosis (Kurt): fourth standardized moment, and it is evaluated as follows:
  $$K=\frac{1}{N}\frac{\sqrt{\sum_{i=1}^{N}{\left(T_{i}-MeanTemp\right)}^{4}}}{{\mathrm{STD}}^{4}}$$
  where *N* is the number of samples.
5. Skewness (Skew): third standardized moment, and it is evaluated as follows:
  $$S=\frac{1}{N}\frac{\sqrt{\sum_{i=1}^{N}{\left(T_{i}-MeanTemp\right)}^{3}}}{{\mathrm{STD}}^{3}}$$
6. 90th percentile (90th P): it is the temperature value below which the 90% of all temperature frequency distribution are comprised.
7. Sample Entropy (*SampEn*): it is defined as the negative natural logarithm of the conditional probability that signals that the subseries of length *m* (pattern length) that match pointwise within a tolerance *r* (similarity factor) also match at the *m* + 1 point. *SampEn* of a time series {*t*_1_,…,*t_N_*} of length *N* is computed employing the following set of equations:
  $$SampEn\left(m,r,N\right)=-\ln\left[\frac{U^{m+1}\left(r\right)}{U^{m}\left(r\right)}\right]\;U^{m}\left(r\right)={\left[N-m\tau \right]}^{-1}\overset{N-m\tau }{\underset{i=1}{\sum }}C_{i}^{m}\left(r\right)\;C_{i}^{m}\left(r\right)=\frac{B_{i}}{N-\left(m+1\right)\tau }\;B_{i}=number\;of\;j\;where\;d\left|T_{i},T_{j}\right|\leq r\;T_{i}=\left(t_{i},t_{i+\tau },\ldots ,t_{i+\left(m-1\right)\tau }\right)\;T_{j}=\left(t_{j},t_{j+\tau },\ldots ,t_{j+\left(m-1\right)\tau }\right)\;i\leq j\leq N-m\tau ,\;j\neq i$$

- Where *U* is the subseries vector considered and *Cm*(*r*) is the probability that any vector *Um*(*j*) is within *r* of *Um*(*i*).
- In this study, *m* = 2 and *r* = 0.2·SD of the signal has been used, in accordance with [39].
- Spatial gradient (Grad): it is evaluated as follow:
-
$\mathrm{Grad}=\nabla T=\frac{\partial T}{\partial x}\hat{ı}+\frac{\partial T}{\partial y}\hat{ȷ}$

- Delta (Δ): difference between the average of the signal in the first 2 s and in the last 2 s of a temporal window of 10 s after the end of the exercise.

To estimate the ARV and MDF from IRI features, the following ML regressors were tested: linear regression (LR), support vector regressor (SVR) with linear and Gaussian kernels, ensemble regressors, and Gaussian process regression (GPR).

The LR is a machine learning algorithm based on supervised learning that performs a regression task, targeting prediction values based on independent variables [40].

The SVR allows the estimation of optimal separating boundaries between data sets by solving a constrained quadratic optimization problem. By the use of different kernel functions, several degrees of nonlinearity can be included in the model [41].

The ensemble regressors use multiple learning algorithms to obtain better performance than those obtained from any of the constituent learning algorithms alone [42].

GPR is a supervised learning method designed to solve regression problems that delivers probabilistic predictions that interpolate the observations [43].

Several ML approaches relying on different kernels and estimation algorithms were tested in order to provide a comprehensive and effective estimation of the relationship between the EMG metrics and IRI features.

In order to estimate ARV and MDF, these models were trained and tested using as input metrics obtained from each ROI separately (i.e., ROI1, ROI2, and ROI3).

The selection of features was conducted using F-tests. Each F-test compares the null hypothesis that the response values grouped by predictor variable values are drawn from populations with the same mean to the alternative hypothesis that the population means differ. A test statistic with a small *p*-value indicates that the corresponding predictor is significant. In addition, in order to assess the generalizability of the procedure’s performance, a nested cross-validation (nCV) was implemented. In nCV, data are divided into folds, and the model is trained iteratively and nestedly on all data with the exception of one fold. In contrast to the outer loop, which estimates the model’s performance across iterations (test), the inner loop determines the optimal hyperparameter (validation). If the number of folds is equal to the number of samples (one fold per sample), then the procedure is known as leave-one-out CV. In this study, the leave-one-subject-out CV was utilized, so the presented results demonstrate an out-of-sample correlation between the *EMG* and IRI, thereby reducing overfitting effects.

## 3. Results

Table 1 reports the correlation analysis between the thermal regressors employed as input to the ML frameworks and the ARV and MDF for each ROI considered.

**Figure 3:**
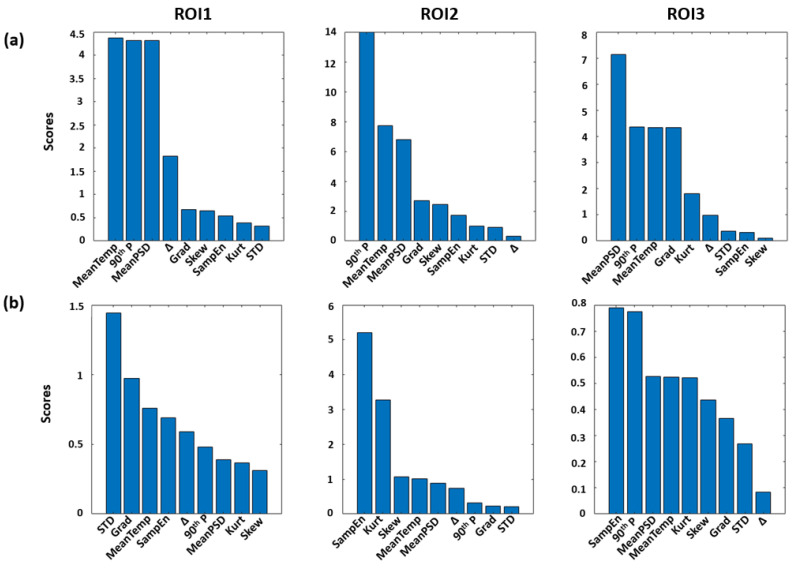
F-tests scores associated to each thermal feature computed for each ROI evaluated for the ARV (**a**) and MDF (**b**) estimation.

Figure 3 shows the results of the features selection procedure. Particularly, Figure 3a shows the scores of the F-tests for the ARV estimation through IRI features, whereas Figure 3b exhibits the scores obtained for the MDF evaluation. Notably, the first three features indicated as most contributive to the prediction of the ARV and MDF were used for further analysis.

Table 2 reports the regression results for each regressor tested for ARV. The performance of the model was evaluated through the root-mean-square error (RMSE) computed on the normalized values (z-score) and the correlation coefficients (*r*) between the EMG-based ARV, considered the gold standard, and the estimated ARV from the IRI features.

Table 3 displays the regression results for each regressor tested for MDF, as measured by the RMSE computed on the normalized values (z-score) and the correlation coefficients between the MDF computed from the EMG signal (the gold standard) and the estimated MDF from the thermal features.

**Figure 4:**
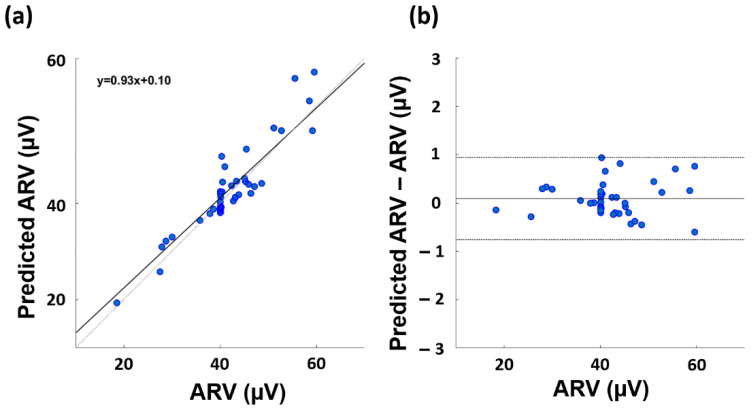
Correlation plot (**a**) and the Bland–Altman plot (**b**) associated to the performance of the Gaussian model developed to estimate the ARV.

Figure 4 displays the correlation plot (Figure 4a) and the Bland–Altman plot (Figure 4b) for the model with the best ARV prediction performance (i.e., the Gaussian model). The paired *t*-test did not assess differences between the gold standard and the predicted variable (t-stat = −1.512; df = 49; *p* = 0.137). The slope of the linear regression equation is 0.93 and the constant term is 0.10.

**Figure 5:**
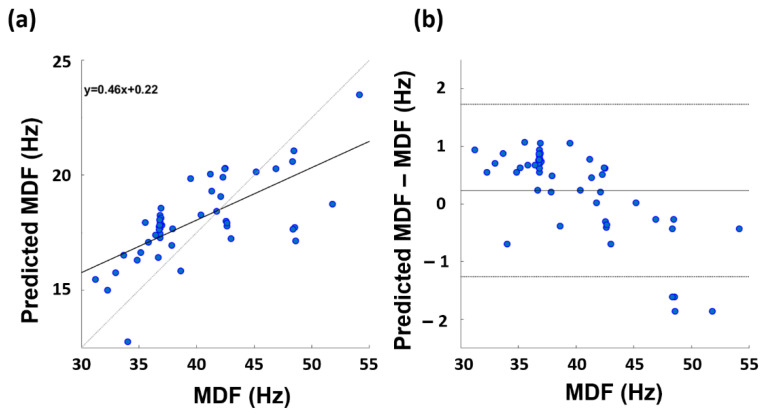
Correlation plot (**a**) and the Bland–Altman plot (**b**) associated to the performance of the Gaussian model developed to estimate the MDF.

Figure 5 depicts the correlation plot and Bland–Altman plot for the model with the most accurate MDF prediction performance. The paired *t*-test assessed differences between the gold standard and the predicted variable (t-stat = −2.165; d.f. = 49; *p* = 0.035). The slope of the linear regression equation is 0.46 and the constant term is 0.22.

## 4. Discussion

This study describes a non-invasive, contactless method for estimating parameters indicative of muscular activity and fatigue using IRI and cross-validated ML approaches. Particularly, the ARV and MDF determined by EMG measurements have been estimated based on thermal characteristics, although the physiological nature of the two processes is different. The sEMG ARV and MDF were chosen as indicators of two distinct aspects of muscle activity: activation level and muscle fatigue. Specifically, the ARV is associated with muscle activity, whereas the MDF reflects muscle fatigue [7]. Regarding IRI metrics, they were chosen as able to provide information regarding the skin temperature oscillations associated to physiological activity. Particularly, both time- and frequency-domain data analysis techniques were used in this study. In addition, non-linear approaches (such as complexity analysis) were employed as they were demonstrated to be able to provide information regarding the activation or dysregulation of a system [44,45]. Moreover, the spatial distribution of temperature within a region of interest is also indicative of changes in superficial blood circulation [46].

Regarding the ARV, the best performance was achieved by the Gaussian model, which provided an estimation with a correlation coefficient of 0.886 and an RMSE on the normalized values of 0.456 when using the thermal metrics evaluated on the ROI3 temperature time course as input for the machine. Hence, in accordance with Cohen’s interpretation of the correlation coefficient [47], a strong correlation between the estimated and measured metric was found. Moreover, the paired *t*-test demonstrated the absence of a bias in the model in estimating the ARV and the Bland–Altman plot showed a correspondence within a tolerance factor of 1.96 standard deviation of the estimated metric with the gold standard. In addition, the linear regression associated with the model showed a slope of 0.93 and a constant term of 0.10. Since a good model should deliver a linear equation with the slope around 1 and the constant term around 0 [48], this result demonstrates the effectiveness of the proposed model in estimating the ARV, as it is computed from the EMG signal.

Considering the thermal features chosen by the features selection procedure, the Grad, *MeanTemp*, and the 90th P selected over the ROI3 delivered the best estimation performance. The correlation analysis demonstrated a large positive correlation between the selected regressors and the ARV.

Notably, the *MeanTemp* and the 90th P were selected also for the ROI2 and ROI1, demonstrating the great contribution of these metrics in the estimation of the ARV.

Concerning the MDF, the best performance was obtained always by the Gaussian model, delivering an estimation characterized by a correlation coefficient of 0.661 and an RMSE on the normalized values of 0.751, when employing as input of the ML framework the thermal metrics evaluated on the ROI3 temperature variations. However, although a good correlation existed between the gold standard and the predicted metric, it should be highlighted that the paired *t*-test assessed the presence of the bias in the estimation of the MDF. Moreover, the Bland–Altman plot showed a systematic error in the estimation of the MDF by the model, which is also visible considering the linear regression equation delivered by the ML approach. It should be stressed that the systematic error and the bias could be corrected in order to improve the estimation. However, further studies are indeed necessary to enlarge the sample size to provide a more generalizable estimation.

The different result and ML predicting performance between amplitude (ARV) and frequency (PDS) descriptors of EMG may be connected to the dissimilar values which ARV and PDS have in depicting muscle fatigue: typically, spectral descriptors exhibiting more consistent variations during fatiguing contractions than amplitude descriptors. This is due to a variety of factors, including changes in conduction velocity during fatigue, the progressive recruitment of larger fibers with greater action potentials, the possible motor-unit rotation during prolonged constant force contractions, and many others. For example, the future study of the changes in fiber conduction velocity during fatiguing contractions may help in better adequate the ML approach to estimate spectral MDF in the fatigued muscle.

Considering the thermal features chosen by the features selection procedure, the *SampEn*, the 90th P, and the *MeanPSD* were selected. The correlation analysis between the regressors and the MDF demonstrated a positive correlation between the 90th P and the *MeanPSD*, and a negative correlation with the *SampEn*. *SampEn* has been shown to decrease with fatigue, especially during concentric contractions. Therefore, the negative correlation observed with IRI features, although not significant, is quite surprising, and deserves further studies. Perhaps extending the correlation study between IRI estimators and complexity estimators by evaluating other different non-linear aspects of muscle fatigue will allow us to indicate which estimators are better approximated by the ML models.

ROI3 delivered the best performance for both the ARV and the MDF among the considered ROIs. The ROI3 was chosen in the region beneath the EMG electrodes. The oscillations in skin temperature are associated with changes in the superficial circulation in response to muscle activity. It is important to note that the best results obtained from ROI3 can be attributed to the anatomy of the muscle fibers, which continues more laterally than below the EMG electrodes. In fact, ROI2, which is lateral in relation to the EMG location and the muscle under consideration, yielded the poorest estimates of EMG parameters. Concerning ROI1, the less-than-optimal results could be attributed to the interference on the superficial temperature caused by the presence of the electrodes, or to the small area examined. In fact, the area between the electrodes is limited, and it is known that accurate temperature measurement through IRI is dependent on the extension of the ROIs. Specifically, small ROIs may produce inaccurate temperature measurements [49].

The proposed procedure could represent a valuable tool for providing some metrics related to muscle fatigue and activity in a non-invasive, contactless manner. Moreover, it should be highlighted that IRI can also provide information on skin injuries, which are associated to the athletes’ physical condition [46,50]. Moreover, the employment of IRI in sport science can allow us to also monitor other physiological signals, such as breathing rate and sweat gland activity, indicative of the psychophysiological condition of the individual [27]. Indeed, previous studies demonstrate that non-contact techniques such as IRI are highly valuable tools to estimate with good accuracy physiological variables (heart rate variability, HRV) using an ML approach [51,52,53].

However, it should be highlighted that the origins of the EMG and IRI signals are different; in fact, the EMG is an electrical signal, whereas the IRI measures thermal effects due to modifications of superficial circulation [54]. Hence, this study aims to assess some correlations between metrics estimated from EMG and IRI features, despite their different natures and the fact that they are indicative of different physiological processes. Notably, establishing correlations between signals of different origins to assess physiological processes is a very common procedure. For instance, functional magnetic resonance imaging (fMRI) [55] or functional near infrared spectroscopy (fNIRS) [56] are able to evaluate the hemodynamic oscillations associated with brain activity, which is an electrical signal evaluated through electroencephalography (EEG) or magnetoencephalography (MEG). As a result, even though the physical origins of functional hemodynamic brain oscillations and electrical depolarizations of neurons differ, it is possible to estimate brain activity from hemodynamics. Importantly, combining these techniques can provide information on the neurovascular coupling, which is impaired in several pathologies, such as Alzheimer’s disease [57]. Hence, combining EMG and IRI could maybe provide some information regarding altered perfusion of the muscle. Another example can be referred to as the evaluation of the HRV through photoplethysmography (PPG). In fact, the HRV is inferred from variations of the heart rhythm, which has an electrical origin, and it is measured through electrocardiography (ECG). However, the modifications of the heart rate produce modifications of the blood volume in the peripheral vessels that could be assessed through PPG. Hence, although the PPG has a different physiological origin with respect to the ECG, the variation of the pulse rate is commonly employed to evaluate HRV metrics [58,59]. Furthermore, remote PPG (rPPG) can assess pulse variations using an RGB video recording with a high-resolution camera [60,61]. Indeed, modifications of the visible optical properties of the skin have a different physiological origin with respect to the heart rate; however, it is possible to estimate HRV metrics from rPPG. Notably, measuring PPG and rPPG does not imply measuring the ECG, but they do allow for the estimation of HRV parameters. 

However, it is important to note that the proposed method does not provide a highly accurate estimate of the MDF metric; therefore, it may be worthwhile to conduct additional research to improve the method’s precision. In fact, the limited number of participants represents the study’s primary limitation. By increasing the sample size, it may be possible to obtain more precise results and to test more intricate machines, such as deep learning. However, it has been showed that ML approaches similar to those used in this study can provide very highly accurate estimations of physiological and performance parameters, and that they are superior to univariate methods [62,63]. Nevertheless, the proposed model was developed using a cross-validation procedure, thereby ensuring its generalization performance.

Importantly, additional research is required to test the capability of estimating other EMG metrics from the skin temperature time course over the activated muscle, considering other ROIs or the entire muscular district of interest during the exercise. In addition, it should be emphasized that only one type of exercise (i.e., bodyweight squats) was analyzed in this study; therefore, additional research should be conducted to examine the relationship between the superficial temperature and the muscle activity in other types of exercise and other muscular districts. Finally, simultaneously with the EMG muscle activation during fatigue, it will be necessary to study the adaptations of the local vessels (for example with locally applied bio-impedance techniques) resulting from the metabolic- and mechanoreceptor-driven activation of the muscles. In this perspective, further studies should investigate the physiology underlying the relationship between the muscles’ temperature and activation also through multimodal approaches. This would allow researchers to investigate the modification of not only the superficial circulation, but also of the deeper tissues perfusion, for instance, through near infrared spectroscopy [64,65].

In this perspective, it should be mentioned that the regulation of the body’s total temperature balance during exercise was not considered in this study. Notably, investigating the regulation of the body’s temperature balance could provide some insight into the physiology of the investigated system, which is beyond the scope of the present study. In fact, the data-driven approach presented in this study only defines relationships between EMG metrics indicating muscle activity and fatigue and skin temperature modulation assessed via infrared thermography, providing a local analysis of the skin temperature rather than a systemic and global investigation. Future studies will indeed be performed to provide insights on the local and systemic physiology of the blood circulation modification associated with muscle activity.

Furthermore, in this study, a fatiguing exercise was chosen to produce an effect on the muscular fatigue measured by EMG, in order to provide variability in the input data set of the various machines tested. Hence, the oscillations in skin temperature can be caused by several physiological factors, including the regulation of the body’s temperature balance and the increase in blood perfusion associated with muscle activity. In fact, it is well documented that unilateral exercise causes skin temperature changes on the untrained side of the body as well, with the trained side always exhibiting a greater thermal response, indicating the presence of both a systemic and local contribution to skin temperature modulations [66,67]. However, it should be noted that the objective of this study was to develop a data-driven model capable of defining a relationship between EMG metrics of muscular activity and fatigue (i.e., ARV and MDF) and the above skin temperature, regardless of the system’s physiology, as typical for a data-driven approach. Importantly, this method is not a substitute for the EMG, as it merely identifies some relationships between muscle activity and skin temperature.

Another concern regarding the feasibility to employ the developed method in sports science is related to the reliability of the IRI measurements. In fact, IRI recordings are highly influenced from both environmental factors and the thermal camera placement with respect to the individual. Concerning the environmental factors, modifications of the measured temperature can be produced by a direct exposition to sunlight and ventilation, and by variations of the ambient temperature and humidity [68,69]. Regarding the placement of the thermal camera, Vardasca et al., 2017, demonstrated that modifications of the participant–thermal camera distance within a range of 20 cm produces non-statistical modifications of the mean temperature evaluated on a ROI, whereas, considering the angles, a range of ±15° can be accepted [49]. The effect of the angle on the IRI measurements for facial regions was also investigated by Cardone et al., 2020, demonstrating that applying a warping procedure, a reliable estimation of regional temperature within a range of head rotation of ±24.23° for yaw and ±13.79° for pitch movements can be obtained [70]. In this perspective, further studies are indeed necessary to evaluate the feasibility to employ the developed method in sports applications during which the distance of the athlete and the thermal camera and the environmental conditions cannot be standardized.

In addition, the developed model can estimate EMG metrics by integrating information over a 10 s time window following muscle activity. To ensure that the thermal signal is not corrupted by motion artifacts, a time window selected immediately after the exercise series was chosen. However, additional research is required to reduce the amplitude of the temporal window for EMG metric prediction. Moreover, it should be noted that during the exercise, the tracking algorithm required to obtain the skin temperature was implemented. Occlusion of the ROIs during the exercise (e.g., due to excessive muscle rotation) could prevent the time course from following the body movement, and motion artifacts could corrupt the signal and render the prediction inaccurate. Therefore, preprocessing methods should be developed to enhance the signal’s quality during movement.

Although preliminary, these findings could pave the way for the use of IRI for contactless evaluation of muscular fatigue and activity metrics in sports science and rehabilitation applications, which could be exploited in an Internet of Things context. Specifically, this model could be suitable for applications in which contact techniques are unsuitable (e.g., individuals with sensitive skin, patients in isolation, newborn incubators, etc.), for the continuous monitoring of athletes’ performance, and for human–machine interaction.

## 5. Conclusions

This study proposed an innovative approach based on Gaussian model to establish relationships between EMG metrics evaluated from vastus medialis and features computed on the above skin temperature time course assessed by IRI. Specifically, ROIs from the muscle were considered to compute thermal features used for the ARV and MDF prediction. The performances of the model were tested through RMSE, correlation analysis, Bland–Altman plot, and paired *t*-test. A model delivering a good estimation of ARV on a temporal window of 10 s was implemented, whereas the model to estimate the MDF exhibited some limitations (i.e., presence of a bias and a systematic error in the prediction) Further studies are indeed necessary to investigate the physiology underlying the link between the muscle activity and the skin temperature. However, although preliminary, these results could pave the way to the employment of IRI for the evaluation of metrics indicative of muscle functioning, suitable for sports science applications or in situations in which contact methods are not implementable.

## Figures and Tables

**Table 1:** Correlation coefficients (*r*) computed between the ARV and MDF and the IRI features used as input to the ML algorithms.

ROI	IRI Feature	*r* (ARV)	*r* (MDF)
1	*MeanTemp*	0.607 (*p* = 0.001)	0.067 (*p* = 0.749)
	STD	−0.213 (*p* = 0.306)	−0.160 (*p* = 0.444)
	Δ	0.202 (*p* = 0.331)	0.240 (*p* = 0.248)
	*MeanPSD*	0.5966 (*p* = 0.002)	0.106 (*p* = 0.615)
	Kurt	−0.167 (*p* = 0.426)	−0.122 (*p* = 0.563)
	Skew	−0.015 (*p* = 0.942)	0.257 (*p* = 0.214)
	90th P	0.5856 (*p* = 0.002)	0.049 (*p* = 0.818)
	*SampEn*	−0.293 (*p* = 0.155)	−0.160 (*p* = 0.446)
	Grad	0.034 (*p* = 0.870)	−0.004 (*p* = 0.986)
2	*MeanTemp*	0.490 (*p* = 0.013)	0.192 (*p* = 0.357)
	STD	−0.052 (*p* = 0.804)	−0.102 (*p* = 0.629)
	Δ	0.256 (*p* = 0.217)	0.264 (*p* = 0.203)
	*MeanPSD*	0.505 (*p* = 0.010)	0.195 (*p* = 0.350)
	Kurt	−0.161 (*p* = 0.441)	−0.257 (*p* = 0.216)
	Skew	0.082 (*p* = 0.699)	0.270 (*p* = 0.192)
	90th P	0.487 (*p* = 0.014)	0.194 (*p* = 0.354)
	*SampEn*	−0.270 (*p* = 0.196)	−0.088 (*p* = 0.675)
	Grad	0.248 (*p* = 0.231)	0.211 (*p* = 0.312)
3	*MeanTemp*	0.541 (*p* = 0.005)	0.196 (*p* = 0.349)
	STD	−0.117 (*p* = 0.579)	−0.151 (*p* = 0.473)
	Δ	0.040 (*p* = 0.849)	−0.064 (*p* = 0.761)
	*MeanPSD*	0.540 (*p* = 0.005)	0.210 (*p* = 0.314)
	Kurt	0.068 (*p* = 0.746)	−0.159 (*p* = 0.449)
	Skew	−0.166 (*p* = 0.427)	0.232 (*p* = 0.264)
	90th P	0.518 (*p* = 0.008)	0.186 (*p* = 0.375)
	*SampEn*	−0.247 (*p* = 0.235)	−0.116 (*p* = 0.581)
	Grad	0.020 (*p* = 0.924)	0.121 (*p* = 0.563)

**Table 2:** Results of the regression obtained by the several ML approaches considered to estimate the ARV from the thermal metrics. Notably, the performance of the model was expressed as root-mean-square error (RMSE) computed on the normalized (z-score) values and the correlation coefficient (*r*) between the gold standard (EMG-based ARV) and the predicted metric (IRI-based ARV). The best model is highlighted with an asterisk.

Model	ROI	RMSE	*r*
LR	1	0.869	0.453 (*p* = 0.023)
SVR (Linear)	1	0.933	0.421 (*p* = 0.036)
SVR (Gaussian)	1	0.959	0.711 (*p* = 6.75·10^−5^)
Ensemble	1	0.824	0.545 (*p* = 0.005)
Gaussian	1	0.902	0.414 (*p* = 0.040)
LR	2	0.926	0.327 (*p* = 0.111)
SVR (Linear)	2	1.004	0.412 (*p* = 0.041)
SVR (Gaussian)	2	0.969	0.555 (*p* = 0.004)
Ensemble	2	0.821	0.550 (*p* = 0.004)
Gaussian	2	0.877	0.451 (*p* = 0.024)
LR	3	1.051	0.377 (*p* = 0.063)
SVR (Linear)	3	0.980	0.356 (*p* = 0.081)
SVR (Gaussian)	3	0.977	0.457 (*p* = 0.022)
Ensemble	3	0.833	0.529 (*p* = 0.007)
Gaussian	3	0.456 *	0.886 (*p* = 3.91·10^−9^) *

**Table 3:** The regression results obtained from the various ML approaches considered for estimating the MDF from thermal metrics. Notably, the model’s performance was expressed as the root mean square error (RMSE) calculated on the normalized (z-score) values and the correlation coefficient (*r*) between the gold standard (EMG-based MDF) and the predicted metric (IRI-based MDF). The best model is highlighted with an asterisk.

Model	ROI	RMSE	*r*
LR	1	1.094	0.368 (*p* = 0.063)
SVR (Linear)	1	1.114	0.271 (*p* = 0.239)
SVR (Gaussian)	1	0.981	0.447 (*p* = 0.025)
Ensemble	1	1.041	0.382 (*p* = 0.059)
Gaussian	1	0.854	0.509 (*p* = 0.009)
LR	2	1.077	0.319 (*p* = 0.121)
SVR (Linear)	2	0.994	0.528 (*p* = 0.007)
SVR (Gaussian)	2	1.016	0.498 (*p* = 0.011)
Ensemble	2	0.993	0.420 (*p* = 0.037)
Gaussian	2	1.017	0.464 (*p* = 0.020)
LR	3	1.085	0.364 (*p* = 0.074)
SVR (Linear)	3	0.775	0.623 (*p* = 8.85·10^−4^)
SVR (Gaussian)	3	1.026	0.335 (*p* = 0.102)
Ensemble	3	1.039	0.442 (*p* = 0.027)
Gaussian	3	0.751 *	0.661 (*p* = 3.21·10^−4^) *

## Data Availability

The data presented in this study are available on request from the corresponding author. The data are not publicly available due to privacy issues.
